# Performance of the Qvella FAST system with downstream application of MALDI-TOF-MS, VITEK 2, and β-LACTA test for same-day identification and antimicrobial susceptibility testing of Gram-negative rods directly from positive blood cultures

**DOI:** 10.1128/spectrum.01328-26

**Published:** 2026-07-14

**Authors:** Jan Esse, Anne-Brit Krappmann, Katja Honold, Johannes Träger, Giuseppe Valenza, Christian Bogdan, Jürgen Held

**Affiliations:** 1 Mikrobiologisches Institut - Klinische Mikrobiologie, Immunologie und Hygiene, Universitätsklinikum Erlangen und Friedrich-Alexander-Universität (FAU) Erlangen-Nürnberg, Erlangen, Germany; 2 FAU Profile Center Immunomedicine, Friedrich-Alexander-Universität (FAU) Erlangen-Nürnberg, Erlangen, Germany; University of California San Diego, La Jolla, California, USA

**Keywords:** blood culture, blood stream infection, sepsis, diagnosis, rapid, antimicrobial susceptibility testing, RAST

## Abstract

Direct identification (ID) and antimicrobial susceptibility testing (AST) of bacteria from positive blood cultures (BCs) are crucial for rapid optimization of therapy in sepsis. The FAST system purifies a Liquid Colony (LC) from BC broth within 25–35 min for downstream applications. We investigated the performance of the FAST system on BCs growing Gram-negative rods combined with mass spectrometry (MALDI-TOF-MS), the β-LACTA-test, and VITEK 2 AST from LC. VITEK 2 AST results were analyzed after 6 and 8 h, and their impact on empiric antibiotic therapy was examined. We included 119 BCs. MALDI-TOF-MS identified all pathogens correctly. The β-LACTA test detected hydrolyzation of third-generation cephalosporins (*n* = 16) within minutes (sensitivity 87.5%, specificity 98.7%). The categorical (99.3%) and essential agreement (99.2%) between VITEK 2 AST from LC and the standard laboratory procedure (VITEK 2 AST from short-term culture) were excellent. However, ampicillin/sulbactam, piperacillin/tazobactam, and moxifloxacin showed very major error rates of 7.3% (*n* = 3), 10.0% (*n* = 1) and 6.3% (*n* = 2), respectively. After 6 h of VITEK 2 incubation, the AST results of some antibiotics were frequently available (ampicillin/sulbactam = 94.5%, ciprofloxacin = 91.7%, cefotaxime = 83.5%), while others were not (piperacillin/tazobactam = 0%, meropenem = 0%). The median time for completion of VITEK 2 AST was 8 h 41 min. Empiric therapy was ineffective in 17% of patients (*n* = 16). The necessity of escalation was first discovered by ID, β-LACTA test and VITEK 2 AST in 2 (12.5%), 7 (43.8%), and 7 (43.8%) patients, respectively. In summary, the FAST system is suitable for use in routine diagnostics. VITEK 2 AST from LC provided same-day results for a substantial proportion of blood cultures that were positive at the start of the laboratory day.

**IMPORTANCE:**

Rapid pathogen identification and antimicrobial susceptibility testing (AST) directly from positive blood cultures are essential to optimize antimicrobial therapy in sepsis. In this study, the FAST system enabled preparation of a Liquid Colony within 25-35 min and provided highly reliable downstream results for Gram-negative bloodstream infections. Combined with MALDI-TOF-MS, β-LACTA testing, and VITEK 2 AST, the workflow achieved excellent agreement with standard laboratory methods while substantially shortening time to actionable results. Same-day AST results were available for a considerable proportion of blood cultures, allowing earlier recognition of ineffective empiric therapy. Importantly, rapid resistance detection by β-LACTA testing and accelerated AST identified the need for escalation of therapy in most inadequately treated patients. These findings demonstrate that integration of the FAST system into routine microbiological diagnostics can support earlier targeted antimicrobial therapy and strengthen antimicrobial stewardship efforts in sepsis management.

## INTRODUCTION

Gram-negative rods are the most common pathogens causing community-acquired and nosocomial bloodstream infections worldwide. In recent years, their proportion has increased (1–3). Timely administration of effective antibiotic therapy is crucial for the survival of patients with sepsis but is becoming increasingly challenging due to the rise of multi-drug-resistant Gram-negative bacteria (MDRGN) (4–8). While effective therapy for the most common Gram-positive bloodstream pathogens (e.g., *Staphylococcus aureus*, *Streptococcus pneumoniae*, *Enterococcus faecalis*, or *Enterococcus faecium*) can be started immediately after identification and rapid detection of individual resistance genes, choosing appropriate treatment for Gram-negative rods is considerably more complex. For this reason, rapid phenotypic antimicrobial susceptibility testing (AST) is an important component of current microbiological diagnostics.

Most rapid AST approaches begin with a positive blood culture (BC) and either perform identification (ID) and/or AST directly from the BC broth (9–13) or purify bacterial cells from it for subsequent downstream standard applications (14–18). One such system is the CE-marked FAST system (Qvella Corporation Europe, Belgium), which enables fully automated preparation of a bacterial Liquid Colony (LC) for up to two positive BCs simultaneously in less than 1 h. Previous studies focused on the comparison of AST results obtained with LC material and with standard laboratory procedures (SLP) utilizing the BD Phoenix system (19–21), broth microdilution (BMD) (22), VITEK 2 (23, 24), MicroScan WalkAway *plus* (25), or disc diffusion testing (DDT) (26). Table S1 provides a review of the respective literature.

In the present study, we investigated the performance of the FAST system combined with matrix-assisted laser desorption/ionization time-of-flight mass spectrometry (MALDI-TOF-MS) for ID, with the β-LACTA test for rapid colorimetric detection of third-generation cephalosporin resistance, and with VITEK 2 for phenotypic AST, and compared it with our SLP. Unlike previous studies, we investigated whether all or part of the AST results could be obtained on the same working day (7 a.m. to 6–8 p.m.) that the BCs were reported positive, and whether these results prompted changes to the empiric antimicrobial therapy.

## MATERIALS AND METHODS

We performed a prospective diagnostic accuracy study at the University Hospital Erlangen, Germany, a tertiary care center with more than 1,400 beds.

**Fig 1:**
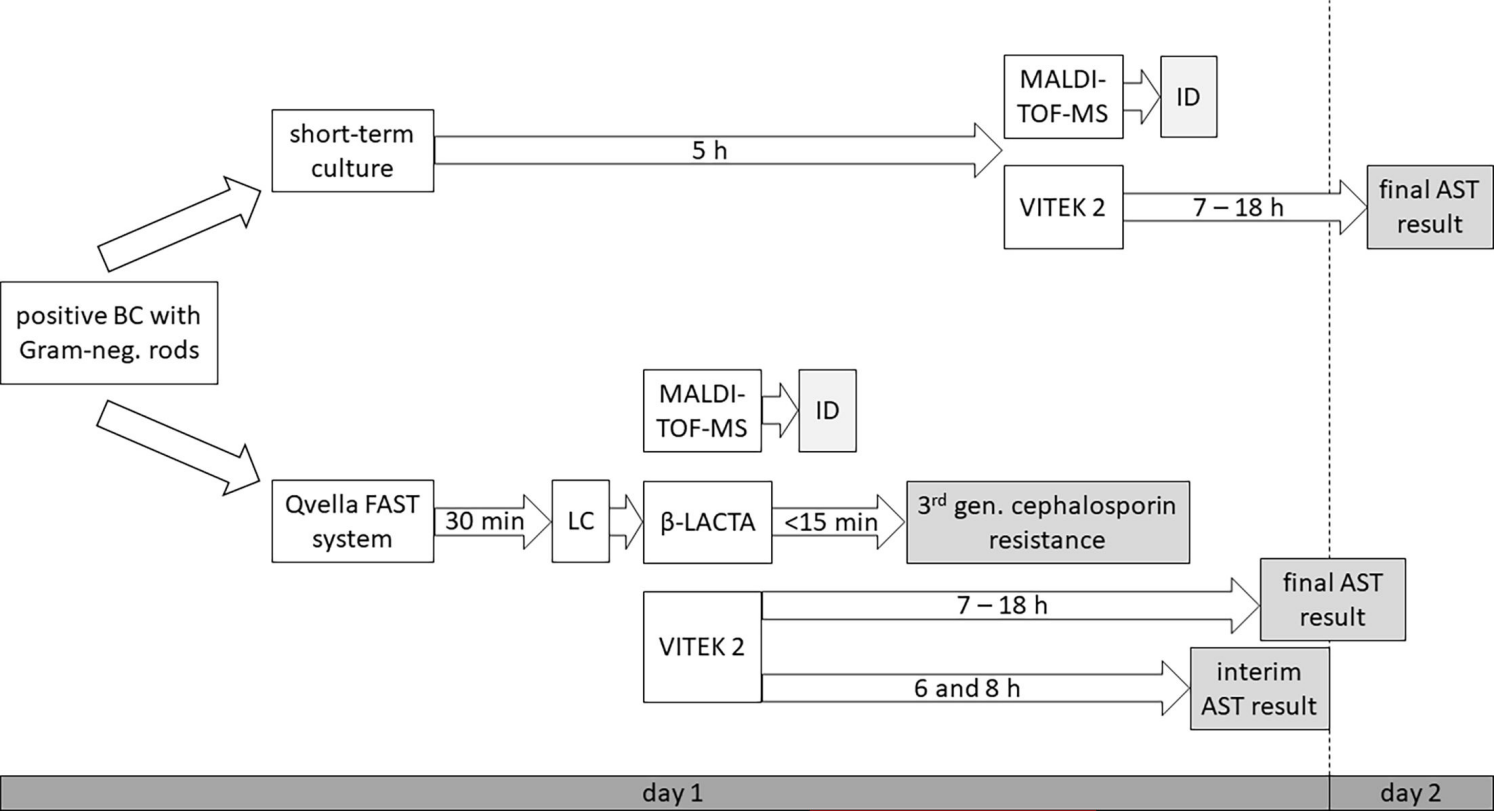
Study procedures. Schematic overview of the procedures in the two study arms: SLP (top) and FAST system (bottom). MALDI-TOF-MS, matrix-assisted laser desorption/ionization time-of-flight mass spectrometry; ID, identification; AST, antimicrobial susceptibility testing; LC, liquid colony; ESBL, extended-spectrum beta-lactamase.

For a period of 5 months (September 2022 to January 2023), the first positive BC of each patient that showed exclusively Gram-negative rods upon Gram-staining and microscopy was included in the study. Mixtures of Gram-negative rods and other pathogens, such as Gram-positive pathogens, were excluded. BACTEC PLUS Aerobic/F and Lytic/10 Anaerobic/F BC bottles were used and incubated in the BACTEC FX BC system (Becton Dickinson GmbH, Germany). The positive BCs were then processed according to our SLP as the reference method and in parallel with the LC workflow (Fig. 1). The SLP consists of short-term culture (4–5 h, 36°C, 5% CO_2_) of a drop of the positive BC broth on Columbia blood agar (scum plate method), subsequent ID by MALDI-TOF-MS (MALDI Biotyper, MBT Compass Library 2023, Bruker Daltonik GmbH, Germany), and automated AST using the VITEK 2 with the AST-N289 card for Enterobacterales and *Acinetobacter* species and the AST-N389 card for *Pseudomonas* species (bioMérieux SA, France). The LC workflow consisted of generating a LC using the FAST system, subsequent ID by MALDI-TOF-MS, colorimetric detection of third-generation cephalosporin resistance by the β-LACTA test (BioRad, France), and AST by VITEK 2. For the FAST system, 1 mL BC broth from the positive BC bottle was transferred to the sample input chamber of the FAST PBC-Prep cartridge, and the fully automated run was started. During centrifugation, the BC broth was mixed with the lysis buffer in the processing chamber, which led to destruction of blood cells. The supernatant was automatically transferred to the waste chamber, and the sediment was washed with buffer and again centrifuged. The cartridge was then unloaded, and the LC (approximately 100 µL) was removed from the processing chamber. Up to two cartridges could be loaded simultaneously into the FAST system. The processing time was approx. 25 min for one cartridge and 35 min for two cartridges. MALDI-TOF-MS ID and VITEK 2 AST were performed according to the manufacturers’ recommendations; however, instead of a solid colony from an agar plate, the more or less viscous LC was used (1 µL per MALDI-TOF target spot and a sufficient amount to generate a bacterial suspension with a McFarland of 0.5 for VITEK 2 AST; no formic acid was used for MALDI-TOF-MS). The β-LACTA test is validated for colonies or preparations from positive BCs of Enterobacterales. It will detect resistance of third-generation cephalosporins due to extended-spectrum β-lactamases (ESBLs), carbapenemases (both serine β-lactamases like *Klebsiella pneumoniae* carbapenemase [KPC] and metallo-β-lactamases), and acquired AmpC β-lactamases because any of these enzymes is able to split the included chromogenic cephalosporin. Acquired penicillinases cannot be detected. It is important to note that, in the event of a positive result, the β-LACTA test is unable to distinguish between the various resistance mechanisms and therefore does not indicate whether the isolate is resistant only to third-generation cephalosporins or also to carbapenems. For the β-LACTA test, the remaining LC (10–50 µL) was mixed with a drop of extraction fluid and a drop of chromogenic solution, and the test was read within 15 min by eye (yellow color: negative; red color: positive; orange color: indeterminate). The results of the β-LACTA test were compared with the AST obtained by SLP as a reference standard. Molecular exclusion of bla_CTX-M-1/-9_ ESBL-genes was performed from colonies by the eazyplex BloodScreen GN loop-mediated isothermal amplification (LAMP) assay (AmplexDiagnostics GmbH, Gars-Bahnhof, Germany), as recommended by the manufacturer.

The AST results of the LC were compared with those of the SLP as follows: if both methods yielded the same ID, LC was considered “correct with high reliability,” if the best MALDI-TOF-MS score was ≥2.0, “correct with medium reliability,” if the best score was between 1.7 and 1.9, and “correct with low reliability,” if the best score was below 1.7. For comparison of AST, results of VITEK 2 AST from LC were assessed after 6 and 8 h of incubation and after the VITEK 2 measurement was completed (usual time to completion of the VITEK 2 panels: approximately 9 h; maximum time to completion: 18 h). AST was evaluated according to the European Committee on Antimicrobial Susceptibility Testing (EUCAST) breakpoint tables version 13.0 (https://www.eucast.org/). Categorical (CA) and essential agreement (EA) between SLP and LC were determined for each antibiotic as recommended by the CLSI Methods Development and Standardization Working Group “Best Practices for Evaluation of Antimicrobial Susceptibility Tests” (27). Briefly, CA was achieved if LC and SLP yielded the same categorical interpretation (susceptible, susceptible upon increased exposure, resistant; note: The EUCAST category “susceptible upon increased exposure” corresponds to the CLSI category “intermediate.” According to EUCAST, AST results can be within an area of technical uncertainty (ATU), where measurements are not reliable and should either not be reported or verified by another test method). A very major error (VME), major error (ME), and minor error (mE) were defined as a “falsely susceptible result”, a “falsely resistant result”, and an error involving the “susceptible upon increased exposure” category, respectively. The CLSI requires that AST systems meet the following conditions: CA ≥ 90%, mE ≤ 10%, ME < 3% and VME < 3%; EA ≥ 90%. The U.S. Food and Drug Administration (FDA), on the other hand, requires a limit of <1.5% for VME. EA was defined as “target” (no minimal inhibitory concentration [MIC] deviation), “range” (MIC deviation of no more than 1 log_2_ dilution), or “out-of-range” (MIC deviation of more than 1 log_2_ dilution). EA was fulfilled for “target” and “range” MIC agreement. AST results without CA were checked by BMD using the MERLIN MICRONAUT system (Bruker Daltonik GmbH, Germany) as recommended by the manufacturer. BMD was performed in triplicates from each bacterial strain, and the median value of the three results was considered the true MIC.

The impact of LC on the antimicrobial therapy was determined by analyzing three time points. As soon as the result of the Gram-staining from the positive BC was available, optimal antibiotic therapy was discussed with the attending physician, a procedure commonly practiced and well established by clinical microbiologists in Germany. The therapy determined at that time was considered to be the “initial therapy.” With the results of the LC available, a potential change in therapy was categorized as “escalation” (i.e., the initially chosen antibiotic was tested resistant), “de-escalation” (i.e., the initially chosen antibiotic was tested susceptible, with the possibility of de-escalation), and “optimal” (i.e., the initially chosen antibiotic was tested susceptible but no possibility of de-escalation). The therapy following receipt of the SLP result was termed “definitive therapy.”

The statistical analyses were performed as two-tailed Fisher’s exact test using SPSS, V29.0.1.0 (IBM, USA). Medians, means, and standard deviation (SD) were calculated with Microsoft Excel 2016 (Microsoft Corporation, USA).

## RESULTS

**Fig 2:**
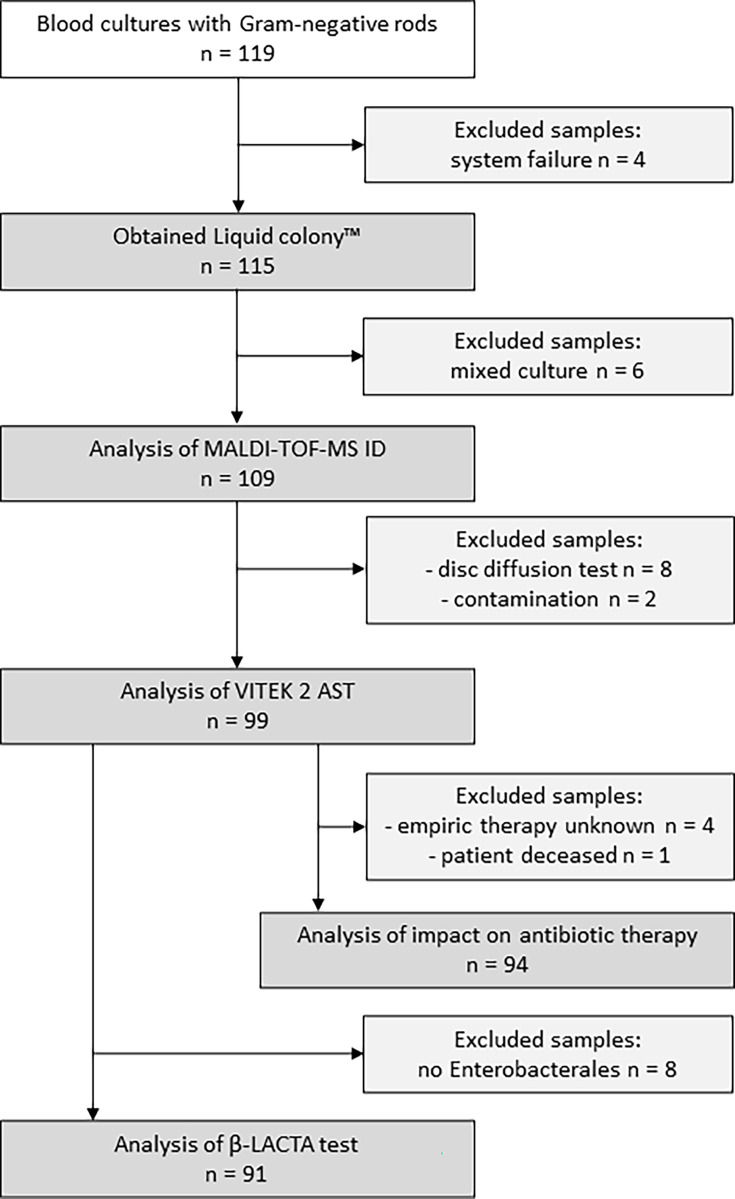
Study flow chart. Study flow chart showing the numbers of analyzed and excluded samples together with the reason for exclusion. *n*, number; MALDI-TOF-MS, matrix-assisted laser desorption/ionization time-of-flight mass spectrometry; ID, identification; AST, antimicrobial susceptibility testing.

Altogether, 119 patients with Gram-negative rods in BCs from routine diagnostics were included in the study. The study flow chart is shown in Fig. 2. Four specimens (3.4%) could not be evaluated because the FAST system aborted the preparation of the LC due to a system failure. Furthermore, six specimens (5.0%) had to be removed from analysis due to growth of more than one pathogen (mixed culture). Thus, the BC samples from 109 patients were eligible for ID by MALDI-TOF-MS. Another eight patients had to be excluded from the subsequent AST analysis because VITEK 2 AST is not recommended or not validated for the detected pathogens (*Acinetobacter spp*. *n* = 2, *Stenotrophomonas maltophilia n* = 1, *Neisseria elongata n* = 1, *Bacteroides fragilis n* = 1, *Capnocytophaga sputigena n* = 1, *Bacteroides ovatus n* = 1, and *Burkholderia gladioli n* = 1). Two cases were interpreted as laboratory contamination, so SLP AST was not performed. Therefore, the samples from 99 patients were eligible for comparison of the VITEK 2 AST results following the LC procedure vs the SLP. Analysis of the β-LACTA results was only possible for Enterobacterales (*n* = 91). With respect to the impact of the LC procedure on antibiotic therapy, data from 94 patients were available, as five patients were excluded due to missing information on initial therapy (*n* = 4) or death before blood culture positivity (*n* = 1).

### Analysis of identification

The detected pathogens and results of MALDI-TOF-MS are listed in Table 1. Among 21 identified species, *Escherichia coli* was the most prevalent, accounting for 56.0% (*n* = 61) of the isolates. Other Enterobacterales accounted for 28.4% (*n* = 31), *Pseudomonas* spp. for 7.3% (*n* = 8), *Acinetobacter* spp. for 1.8% (*n* = 2), and miscellaneous Gram-negative bacteria for 6.4% (*n* = 7) of the cases. MALDI-TOF-MS performed from LC correctly identified all strains at the species level, with high reliability in 95 strains (87.1%), medium reliability in 11 strains (10.1%), and low reliability in three strains (2.8%). The mean MALDI-TOF-MS score obtained using the SLP (2.28; SD 0.14; range, 1.86–2.56) was significantly higher (*P* < 0.01) than that obtained using the LC procedure (2.20; SD 0.20; range, 1.40–2.50). Table S2 summarizes the results for strains with MALDI-TOF MS scores below 2.0. *Capnocytophaga sputigena*, *Escherichia coli*, and *Burkholderia gladioli* were identified from LC with scores of 1.40, 1.48, and 1.68, respectively. Among the top 10 ID results obtained with MALDI-TOF-MS, *Capnocytophaga sputigena* appeared six times, *Escherichia coli* appeared seven times, and *Burkholderia gladioli* appeared five times, suggesting that despite the low scores, these IDs were correct. The same applied to the results of strains with MALDI-TOF-MS scores between 1.7 and 2.0, where the correct ID appeared 6 to 10 times among the top 10 ID results.

**TABLE 1:** Spectrum of tested pathogens and reliability of MALDI-TOF-MS ID results^*a*^

Species	Standard laboratory procedure (SLP)			Liquid colony (LC)		
	ID (no. of strains) (%)	Mean MALDI-TOF-MS score (SD)	Reliability of MALDI-TOF-MS (high/medium/low)	ID matching the SLP ID (no. of strains) (%)	Mean MALDI-TOF-MS score (SD)	Reliability of MALDI-TOF-MS (high/medium/low)
Total	109 (100.0)	2.28 (0.14)	105/3/0^*b*^	109 (100)	2.20 (0.20)	95/11/3
*Escherichia coli*	61 (56.0)	2.30 (0.12)	59/1/0^*b*^	61 (100)	2.23 (0.17)	56/4/1
*Klebsiella pneumoniae*	15 (13.8)	2.30 (0.14)	15/0/0	15 (100)	2.28 (0.12)	15/0/0
*Pseudomonas aeruginosa*	7 (6.4)	2.26 (0.17)	6/1/0	7 (100)	2.25 (0.10)	7/0/0
*Enterobacter cloacae complex*	4 (3.7)	2.15 (0.21)	3/1/0	4 (100)	2.06 (0.21)	3/1/0
*Klebsiella oxytoca*	3 (2.8)	2.23 (0.17)	3/0/0	3 (100)	2.32 (0.11)	3/0/0
*Serratia marcescens*	2 (1.8)	2.18 (0.04)	2/0/0	2 (100)	2.00 (0.05)	1/1/0
*Acinetobacter species*	2 (1.8)	2.14 (0.12)	2/0/0	2 (100)	1.98 (0.28)	1/1/0
*Klebsiella aerogenes*	2 (1.8)	2.07 (0.10)	2/0/0	2 (100)	2.20 (0.10)	2/0/0
*Proteus mirabilis*	1 (0.9)	2.47 (n.a.)	1/0/0	1 (100)	2.47 (n.a.)	1/0/0
*Pseudomonas putida – group*	1 (0.9)	2.27 (n.a.)	1/0/0	1 (100)	1.94 (n.a.)	0/1/0
*Citrobacter freundii*	1 (0.9)	2.08 (n.a.)	1/0/0	1 (100)	2.06 (n.a.)	1/0/0
*Citrobacter koseri*	1 (0.9)	2.46 (n.a.)	1/0/0	1 (100)	2.36 (n.a.)	1/0/0
*Stenotrophomonas maltophilia*	1 (0.9)	2.09 (n.a.)	1/0/0	1 (100)	1.91 (n.a.)	0/1/0
*Neisseria elongata*	1 (0.9)	2.19 (n.a.)	1/0/0	1 (100)	2.04 (n.a.)	1/0/0
*Bacteroides fragilis*	1 (0.9)	2.49 (n.a.)	1/0/0	1 (100)	2.15 (n.a.)	1/0/0
*Capnocytophaga sputigena*	1 (0.9)	2.50 (n.a.)	1/0/0	1 (100)	1.40 (n.a.)	0/0/1
*Bacteroides ovatus*	1 (0.9)	2.02 (n.a.)	1/0/0	1 (100)	1.79 (n.a.)	0/1/0
*Moraxella osloensis*	1 (0.9)	2.04 (n.a.)	1/0/0	1 (100)	1.82 (n.a.)	0/1/0
*Morganella morganii*	1 (0.9)	2.45 (n.a.)	1/0/0	1 (100)	2.34 (n.a.)	1/0/0
*Burkholderia gladioli*	1 (0.9)	2.22 (n.a.)	1/0/0	1 (100)	1.68 (n.a.)	0/0/1
*Raoultella ornitholytica*	1 (0.9)	2.43 (n.a.)	1/0/0	1 (100)	2.36 (n.a.)	1/0/0

^*a*^ Reliability of MALDI-TOF-MS was considered “high,” if the best MALDI-TOF-MS score was ≥2, “medium,” if the best score was between 1.7 and 1.9, and “low,” if the best score was below 1.7.

^*b*^ Documentation of *Escherichia coli *is lacking one MALDI-TOF-MS score. ID, identification; no., number; SD, standard deviation; n.a., not applicable.

### β-LACTA test from LC

Resistance against third-generation cephalosporins was detected within less than 15 min by the β-LACTA test from LC with a sensitivity and specificity of 87.5% or 98.7%, respectively (Table S5). False-negative test results occurred with one strain of *Enterobacter cloacae* complex and of *Klebsiella aerogenes*, while a false-positive result occurred with one strain of *Klebsiella oxytoca*. All true β-LACTA test-positive isolates were found to carry a CTX-M1/9 ESBL, as confirmed by the eazyplex BloodScreen GN LAMP assay. In the two false-negative strains, no CTX-M1/9 ESBL could be detected, and their resistance phenotype was most likely attributable to AmpC β-lactamase production (Table S6).

### Agreement of VITEK AST

Overall, 99 specimens were included in this part of the study. Results for CA and EA are found in Tables 2 and 3. The overall CA (99.3%), mE rate (0.2%), and ME rate (0%) of all antibiotics met the requirements specified by the CLSI. However, VME rates above CLSI limits were observed for piperacillin/tazobactam (*n* = 1; 10.0%), ampicillin/sulbactam (*n* = 3; 7.3%), and moxifloxacin (*n* = 2; 6.3%). Overall VME rate (*n* = 6, 2.6%) fulfilled the CLSI requirements but exceeded the FDA requirements. Analysis of the six VME cases revealed that in five cases, the LC-MIC was only one dilution step below the SLP-MIC, but since the EUCAST does not define a category of “susceptible at increased exposure” for these antibiotics, this minor difference already led to VMEs (Table 4). Accordingly, the EA between LC and SLP fulfilled CLSI and FDA limits in all antibiotics (overall EA 99.2%). Table S4 contains the MIC deviations between SLP and LC.

**TABLE 2:** Categorical agreement of VITEK 2 AST results comparing liquid colony and standard laboratory procedure^*a*^

Antibiotic	Categorical agreement (no. of CA/total no. of results) (%)	Minor error (no. of mE/total no. of results) (%)	Major error (no. of ME/total no. of susceptible results) (%)	Very major error (no. of VME/total no. of resistant results) (%)	No. of results in ATU (no. in ATU/total no. of results) (%)
Total	1261/1270 (99.3)	3/1270 (0.2)	0/931 (0.0)	**6/227** (**2.6)^*e*^**	5/196 (2.6)
Ampicillin	91/91 (100)	0/91 (0.0)	0/25 (0.0)	0/66 (0.0)	n.a.
Ampicillin/sulbactam	88/91 (96.7)	0/91 (0.0)	0/50 (0.0)	**3/41** (**7.3)^*e*^**	n.a.
Piperacillin/tazobactam^*b*^	93/94 (98.9)	0/94 (0.0)	0/79 (0.0)	**1/10** (**10.0)^*e*^**	1/97 (1.0)
Cefuroxime	80/80 (100)	0/80 (0.0)	n.a.	n.a.	n.a.
Cefotaxime^*b*^	91/91 (100)	0/91 (0.0)	0/75 (0.0)	0/16 (0.0)	n.a.
Ceftazidime	98/99 (99.0)	1/99 (1.0)	0/77 (0.0)	0/8 (0.0)	n.a.
Meropenem^*c*^	97/98 (99.0)	1/98 (1.0)	0/97 (0.0)	0/1 (0.0)	n.a.
Gentamicin	91/91 (100)	0/91 (0.0)	0/83 (0.0)	0/8 (0.0)	n.a.
Tobramycin	99/99 (100)	0/99 (0.0)	0/91 (0.0)	0/8 (0.0)	n.a.
Ciprofloxacin	92/93 (98.9)	1/93 (1.1)	0/75 (0.0)	0/12 (0.0)	4/99 (4.0)
Moxifloxacin	89/91 (97.8)	0/91 (0.0)	0/59 (0.0)	**2/32** (**6.3)^*e*^**	n.a.
Trimethoprim/sulfamethoxazole	91/91 (100)	0/91 (0.0)	0/69 (0.0)	0/22 (0.0)	n.a.
Tigecycline^*d*^	62/62 (100)	0/62 (0.0)	0/61 (0.0)	0/1 (0.0)	n.a.
Imipenem	99/99 (100)	0/99 (0.0)	0/90 (0.0)	0/2 (0.0)	n.a.

^*a*^ Categorical agreement was achieved if the AST method under evaluation (i.e., liquid colony) and the reference method (i.e., standard laboratory procedure) yielded the same categorical interpretation. Very major error, major error, and minor error are defined as a “falsely susceptible result,” a “falsely resistant result” and an error involving the “susceptible upon increased exposure” category, respectively.

^*b*^ Two measurement errors, EUCAST breakpoints for cefuroxime only applicable for *Escherichia coli*, *Klebsiella* spp. (except for Klebsiella aerogenes), *Raoultella* spp., and *Proteus mirabilis*; EUCAST breakpoints for cefuroxime only applicable for *Escherichia coli*, *Klebsiella *spp. (except for *Klebsiella aerogenes*), *Raoultella *spp., and *Proteus mirabilis*.

^*c*^ One measurement error; measurement errors were due to partially aborted VITEK test run.

^*d*^ EUCAST breakpoints for tigecycline only applicable for *Escherichia coli *and *Citrobacter koseri*, FDA requirements not fulfilled (bold).

^*e*^ CLSI and FDA requirements not fulfilled (bold); No., number; ATU, area of technical uncertainty; n.a., not applicable.

**TABLE 3:** Essential agreement of VITEK 2 AST results comparing Liquid colony and standard laboratory procedure^*a*^

Antibiotic	Essential agreement (no. of EA/total no.) (%)	“Target” agreement (no. of TE/no.) (%)	“Range” agreement (no. of RE/total no.) (%)	“Out-of-range” agreement (no. of OR/total no.) (%)
Total	1,269/1,279 (99.2)	1,209/1,279 (94.5)	60/1,279 (4.7)	10/1,279 (0.8)
Ampicillin	91/91 (100)	82/91 (90.1)	9/91 (9.9)	0/91 (0.0)
Ampicillin/sulbactam	90/91 (98.9)	70/91 (76.9)	20/91 (21.9)	1/91 (1.1)
Piperacillin/tazobactam^*b*^	94/97 (96.9)	90/97 (92.8)	4/97 (4.1)	3/97 (3.1)
Cefuroxime^*c*^	80/80 (100)	75/80 (93.8)	5/80 (6.2)	0/80 (0.0)
Cefotaxime	89/91 (97.8)	87/91 (95.6)	2/91 (2.2)	2/91 (2.2)
Ceftazidime	98/99 (99.0)	96/99 (97.0)	2/99 (2.0)	1/99 (1.0)
Meropenem^*d*^	97/98 (99.0)	96/98 (98.0)	1/98 (1.0)	1/98 (1.0)
Gentamicin	91/91 (100)	91/91 (100)	0/91 (0.0)	0/91 (0.0)
Tobramycin	99/99 (100)	96/99 (97.0)	3/99 (3.0)	0/99 (0.0)
Ciprofloxacin	98/99 (99.0)	93/99 (93.9)	5/99 (5.1)	1/99 (1.0)
Moxifloxacin	91/91 (100)	88/91 (96.7)	3/91 (3.3)	0/91 (0.0)
Trimethoprim/sulfamethoxazole	91/91 (100)	90/91 (98.0)	1/91 (1.1)	0/91 (0.0)
Tigecycline^*e*^	62/62 (100)	62/62 (100)	0/62 (0.0)	0/62 (0.0)
Imipenem	98/99 (99.0)	93/99 (93.9)	5/99 (5.1)	1/99 (1.0)

^*a*^ Essential agreement was defined as “target” (no MIC deviation), “range” (MIC deviation of no more than 1 log² dilution) or “out-of-range” (MIC deviation of more than 1 log² dilution). Both target and range fulfilled the essential agreement (gray shaded area).

^*b*^ Two missing results due to partially aborted VITEK test run.

^*c*^ EUCAST breakpoints for cefuroxime only applicable for *Escherichia coli*, *Klebsiella* spp. (except for Klebsiella aerogenes), *Raoultella *spp., and *Proteus mirabilis*.

^*d*^ One missing result due to partially aborted VITEK test run.

^*e*^ EUCAST breakpoints for tigecycline only applicable for *Escherichia coli *and *Citrobacter koseri*; No., number; EA, essential agreement; TE, target agreement; RE, range agreement; OR, out-of-range agreement.

**TABLE 4:** Categorical error analysis

Study no.	Pathogen	Antibiotic	Categorical error	MIC VITEK 2 from LC (mg/L)	MIC VITEK 2 from SLP (mg/L)	MIC BMD (mg/L)	Categorical result confirmed by BMD	Time to result VITEK 2 from LC (hh:mm)	Time to result SLP (hh:mm)
19	*Proteus mirabilis*	Moxifloxacin	VME	≤0.25	0.5	0.5	SLP	15:46	15:33
26	*Escherichia coli*	Ampicillin/sulbactam	VME	8	16	>16	SLP	10:52	10:10
31	*Escherichia coli* ^*a*^	Piperacillin/tazobactam	VME	≤4	≥128	>128	SLP	07:27	13:20
32	*Klebsiella pneumoniae* ^*a*^	Ampicillin/sulbactam	VME	8	16	16	SLP	09:10	08:41
41	*Serratia marcescens*	Moxifloxacin	VME	≤0.25	0.5	0.5	SLP	14:19	14:49
112	*Escherichia coli*	Ampicillin/sulbactam	VME	8	16	>16	SLP	07:36	07:28
85	*Pseudomonas* species	Ceftazidime	mE	16	1	2	SLP	17:43	16:45
85	*Pseudomonas* species	Ciprofloxacin	mE	2	≤0.06	0.125	SLP	17:43	16:45
122	*Pseudomonas* aeruginosa	Meropenem	mE	4	2	1	SLP	11:40	12:07

^*a*^ VME clinically relevant because necessary escalation of antimicrobial therapy was not identified. MIC, minimum inhibitory concentration; LC, liquid colony; SLP, standard laboratory procedure; BMD, broth microdilution. Very major error, major error, and minor error are defined as a falsely susceptible result, a falsely resistant result and an error involving the susceptible upon increased exposure category, respectively.

### Time to result of VITEK 2 AST and possibility of same-day AST results

There was no significant difference (*P* = 0.943) between the mean time for completion of VITEK 2 AST from LC (9 h 20 min, SD: 2 h 11 min; median 8 h 41 min, range 7 h 13 min–17 h 43 min) and SLP, i.e., VITEK 2 AST from colony material (mean 9 h 21 min, SD: 2 h 18 min, median 8 h 30 min, range 7 h 13 min–17 h 45 min). However, since the start of the SLP-VITEK 2 AST was delayed by 4.5 h due to the necessity of short-term incubation, the VITEK 2 AST from LC had a time advantage. When the laboratory closes at 6 p.m. and 8 p.m., 75 (76%) and 87 (88%) of the VITEK 2 ASTs from LC of BCs that were positive at laboratory opening would have been completed. For comparison, no SLP-VITEK 2 AST would have been completed by 6 p.m., and 30 (30%) would have been completed by 8 p.m., respectively. For BCs that turned positive after 10 a.m. or 12 p.m., even VITEK 2 ASTs from LC would never have been completed.

The VITEK 2 system compares the individual growth curves of each antibiotic with those stored in its database and reports the results as soon as a clear match exists. This means that the MIC of individual antibiotics can be viewed before the entire AST has been completed. For this reason, we analyzed the available VITEK 2 AST results from LC after 6 and 8 h of incubation. After 6 h of incubation, the results for some antibiotics were frequently available (ampicillin/sulbactam 94.5%, ciprofloxacin 91.7%, cefotaxime 83.5%, and ceftazidime 76.0%), while for others no results were provided by the system at this time point (piperacillin/tazobactam, meropenem) (Table S3). However, because susceptibility to ampicillin/sulbactam (59.3% of cases susceptible at 6 h) usually also implies susceptibility to piperacillin/tazobactam, 56.0% of piperacillin/tazobactam results were indirectly available after 6 h of incubation. At the 8-h time point, 17.4% of piperacillin/tazobactam results and 69.6% of meropenem results were regularly available. Together with the extrapolated results from ampicillin/sulbactam susceptibility, 77.4% of piperacillin/tazobactam results were available after 8 h.

### Therapeutic implications

The results obtained with 94 of the specimens were eligible to assess the therapeutic impact of the FAST system. Most patients initially received piperacillin/tazobactam (*n* = 67, 71.3%), meropenem (*n* = 13, 13.8%), or ampicillin/sulbactam (*n* = 4, 4.3%) monotherapy, while five patients (5.3%) were additionally treated with ciprofloxacin (*n* = 3), tobramycin (*n* = 1), or clindamycin (*n* = 1).

In 16 patients (17.0%), an escalation of the initial antibiotic therapy was required, whereas the chosen empiric antibiotic therapy could have been de-escalated in 43 patients (45.7%) and was already optimal in 35 patients (37.2%). The VITEK 2 results obtained with LC samples correctly identified 14 of 16 necessary escalations (87.5%) and all possible de-escalations. In the two cases where the VITEK 2 analysis of the LC samples missed the necessary escalation, ampicillin/sulbactam (MIC from LC: 8 mg/L; MIC from SLP and MBD: 16 mg/L) and piperacillin/tazobactam (MIC from LC: ≤4 mg/L; MIC from SLP and MBD: ≥128 mg/L) were measured falsely susceptible and should have been replaced by piperacillin/tazobactam or meropenem, respectively. While the MIC value of the first VME differed only by one dilution step, the second VME showed a maximum difference between both methods. Another striking feature of the second case was that the time required to complete the VITEK AST was only 7 h 27 min for the test from LC compared with 13 h 20 min for the SLP test.

The β-LACTA test performed with LC samples identified in all cases (*n* = 8) the necessary escalation to meropenem due to resistance against third-generation cephalosporins. In one case, however, this escalation would have been ineffective because the identified pathogen (*Klebsiella pneumoniae*) produced a New Delhi metallo-β-lactamase (NDM).

## DISCUSSION

We prospectively evaluated the performance of the FAST system in combination with MALDI-TOF-MS, the β-LACTA test, and VITEK 2 AST and compared it to our SLP for positive BCs with growth of Gram-negative rods.

We decided to study only Gram-negative rods, as targeted antibiotic treatment of Gram-positive cocci is already possible with sufficient certainty after detection of the *mecA/C* gene in staphylococci or identification of streptococci and enterococci. For this purpose, quick and inexpensive tests are available that can be performed directly from positive BCs (e.g., the eazyplex system [AmplexDiagnostics GmbH, Germany] or the Molecular Mouse [Alifax S.r.l., Italy]). However, for Gram-negative rods, such systems can usually only determine the existence of a limited number of genes for extended spectrum β-lactamases (ESBLs) or carbapenemases and therefore phenotypic AST is still mandatory for targeted antibiotic treatment.

In Germany and many other European countries, microbiological laboratories are not open around the clock, but usually have opening hours between 7 a.m. and 6 or 8 p.m. As a result, all BCs that are reported as positive by the automated BC incubation systems during closing times will only be processed further after the diagnostic laboratory reopens in the morning. The usual SLP is a short-term culture for 5 h (scum plate method) and subsequent ID by MALDI-TOF-MS followed by some kind of phenotypic AST. Accordingly, the AST will be started around 1 p.m., i.e., 5 to 7 h before the end of the laboratory day. However, this time is not sufficient for automated AST systems, such as VITEK 2 to complete their measurements, so AST results are usually not available until the next day. In order to obtain AST results on the day of BC positivity, it would be necessary to shorten the time of the first incubation or to use a faster AST method. In our study, we used the FAST system to generate a LC within approximately 30 min. This LC was then used for further applications.

In terms of reliability, the FAST system proved to be robust. Only four samples (3.4%) could not be processed due to a system failure, which represents an error rate similar to most previous studies (Table S1). Only Kuo et al. (19) reported a significantly higher error rate (16.5%, *P* ≤ 0.001) and Verroken et al. (20) reported a significantly lower error rate (0.4%, *P* = 0.03). The reasons for these differences are unclear, but unlike our procedure, both studies partially included spiked control BCs in their workflow.

The ID of Gram-negative rods from LC using MALDI-TOF-MS worked exceptionally well. All isolates were correctly identified and about 4.5 h earlier than by the SLP. However, the reliability of the ID, expressed via the MALDI-TOF-MS score, was significantly better with SLP. In total, 87.1% of pathogens were identified from LC with a score of 2.0 or more, which is reliable at the species level according to the manufacturer. This rate is slightly lower than in comparable studies (Table S1). However, another 10.1% of pathogens were identified with a score between 1.7 and 2.0, which is reliable at the genus level. However, a look at the ten best-matching IDs from these measurements showed that between six to 10 of them were identical to the top-ranked species. Even in the case of the three strains with a MALDI-TOF-MS score below 1.7, the top 10 IDs listed 5, 6, or 7 identical species, respectively. In routine clinical practice, this would be acceptable for a preliminary identification at the species level, which could easily be verified the next day by MALDI-TOF-MS using a conventional colony. It has to be noted that our results apply only to the MALDI Biotyper and that it is not possible to evaluate the score and the top ten hits using systems, such as the VITEK MS. The comparison of our results with those of previous studies showed that their ID agreement at the genus level was similar to ours, ranging from 90.6% to 100% (Table S1).

The additional use of the β-LACTA test from LC provided useful information about the resistance of Enterobacterales to third-generation cephalosporins, with a positive result usually visible after less than 5 min. Both sensitivity and specificity were over 95% and therefore comparable to the study by Verroken et al. (sensitivity 95.8%, specificity 100%) (20). Resistance against third-generation cephalosporins was missed in one *Enterobacter cloacae* complex strain and one *Klebsiella aerogenes* strain. Following the molecular exclusion of CTX-M1/9-ESBL (Table S6), the resistance phenotype is most likely attributable to AmpC β-lactamases production. Although the manufacturer claims that the β-LACTA test can detect acquired AmpC β-lactamases, a review of the literature revealed that this does not seem to be the case. Parmeland et al., as well as Durand et al., tested 50 Enterobacterales strains with AmpC hyperproduction, including *Enterobacter cloacae* and *Klebsiella aerogenes* isolates, and all were falsely negative in the β-LACTA test (28, 29). Furthermore, all β-LACTA test-positive isolates in our study were confirmed to be CTX-M1/9-ESBL-positive. Another problem with the β-LACTA test is that, in the event of a positive result, it cannot distinguish whether the isolate is resistant only to third-generation cephalosporins or also to carbapenems. That is why the escalation from piperacillin/tazobactam to meropenem in one patient with NDM-positive *Klebsiella pneumoniae* would have been ineffective. However, this limitation could be addressed by performing the β-CARBA test from the same manufacturer—which detects resistance to carbapenems—following a positive β-LACTA result.

The comparison of the VITEK AST results obtained with LC samples or the SLP showed that the overall EA was very good (99.2%), and CLSI and FDA limits were fulfilled for every antibiotic, which is in accordance with prior studies (Table S6). However, although the overall CA was similarly good (99.3%), high VME rates were observed for ampicillin/sulbactam (7.3%, *n* = 3) and piperacillin/tazobactam (10.0%, *n* = 1), exceeding the CLSI and FDA limits. In contrast, our SLP correctly measured the susceptibility against these antibiotics. The VME rate of 10% for piperacillin/tazobactam is particularly worrisome, as empirical therapy at many hospitals including ours recommends the administration of this antibiotic in cases of possible Gram-negative sepsis. However, it should be noted that piperacillin/tazobactam-resistant isolates were very rare in our study (*n* = 10), and the measured VME rate of 10% results from only one falsely susceptible isolate. The MIC value obtained with LC of this isolate was ≤4 mg/L compared with ≥128 mg/L from SLP and BMD. Additionally, VITEK AST took only 7 h 27 min with the LC sample but 13 h 20 min using the SLP, a time difference never seen with other isolates. This shows that something was fundamentally wrong with the testing of this isolate and even a sample mix-up must be taken into consideration. A detailed comparison of the AST results for piperacillin/tazobactam achieved with LC vs SLP samples showed that 93% of the isolates (*n* = 90) had exactly the same MIC and another 4% (*n* = 4) had a MIC difference ±1 dilution step (Table S4). Thus, AST from LC was clearly reliable for piperacillin/tazobactam. For ampicillin/sulbactam, all three VMEs belonged to isolates where there was only one MIC dilution step between the LC and the SLP result. However, since the EUCAST does not define a category of “susceptible at increased exposure” for ampicillin/sulbactam, this minor difference already led to a VME. Such VMEs of ampicillin/sulbactam were also reported from other studies (20, 24); however, they did not exceed the CLSI limit and only in one case the FDA limit for VMEs (24).

A main objective of our study was to investigate whether same-day results were available with VITEK 2 AST from LC. Overall, our results showed that when the laboratory closes at 6 p.m. and 8 p.m., 76% and 88 % of the VITEK 2 ASTs from the BCs that are positive at laboratory opening would have been completed. However, BCs that turned positive after 10 a.m. or 12 p.m. would never have been fully processed. Nevertheless, a substantial proportion of results were already available after six h: more than 90% for ampicillin/sulbactam, tigecycline, quinolones, and aminoglycosides, and more than 75% for ampicillin and cephalosporins. Unfortunately, the results for the most frequently used empirical antibiotics, piperacillin/tazobactam and meropenem, were never provided by the VITEK 2 at this early time point. In the case of ampicillin/sulbactam susceptibility, this result could be extrapolated to piperacillin/tazobactam, which was possible in 56.0% of the isolates. At the 8-h time point, 69.6% of meropenem results and 77.4% of piperacillin/tazobactam results (including the extrapolated results) were available. Overall, this means that in a considerable number of patients, useful information on the efficacy of empiric antibiotic therapy is available for all BCs that turn positive until noon. It has to be noted that the preliminary evaluation of single VITEK 2 results is not FDA cleared. However, VITEK 2 MIC results usually do not change once they are displayed; at most, the clinical efficacy may be adjusted by the VITEK expert system based on intrinsic resistance or suspected phenotypes. Additionally, the procedure to infer a susceptible result for ampicillin/sulbactam to piperacillin/tazobactam is not explicitly recommended by EUCAST or CLSI. However, piperacillin usually has better activity against Enterobacteriaceae than ampicillin, and tazobactam is a more potent inhibitor of most beta-lactamases (e.g., TEM, SHV, ESBL) than sulbactam (30). An analysis of our routine AST results for Enterobacteriaceae over the past 10 years (n ≈ 70,000) revealed that only 0.08% exhibited a phenotype in which they were susceptible to ampicillin/sulbactam but resistant to piperacillin/tazobactam. Furthermore, this practice involves the risk that errors observed in ampicillin/sulbactam testing (three VMEs in our study) may be transferred to piperacillin/tazobactam. Nevertheless, we are convinced that such an assumption is acceptable and reasonable for the early adjustment of antimicrobial therapy.

Empirical therapy should cover the vast majority of pathogens that are likely causing the infection in a severely ill patient. To evaluate the impact of rapid AST methods, it is also important to know in how many patients the AST result has a therapeutic consequence. In our study, empiric therapy was optimal in 37% of patients and effective in another 46% of patients. This means that 17% of patients (*n* = 16) received ineffective antibiotic therapy. In two patients, an escalation from ampicillin/sulbactam to piperacillin/tazobactam was necessary as the bloodstream infection was caused by *Pseudomonas aeruginosa*. However, the necessity of escalation had already become apparent based on the MALDI-TOF-MS identification from LC because ampicillin/sulbactam is intrinsically resistant in *Pseudomonas aeruginosa*. In another seven patients, an escalation from piperacillin/tazobactam to meropenem was necessary because the pathogens produced an ESBL. Again, this escalation had already been detected by the β-LACTA test. In total, ineffective empirical therapy was identified by VITEK 2 AST using LC samples in only seven patients (7.4%). In addition, diagnosis of an inadequate antimicrobial therapy on the same day would require that the BCs were already positive in the morning. However, this seemingly low impact of VITEK 2 AST from LC is highly dependent on the prevalence of MDRGN. Thus, our data may not be applicable to regions with high resistance rates.

A limitation of our study is the rather small sample size, even though the number of Gram-negative rods examined by us (109 strains) is similar to previous studies. The small sample size, in combination with a low MDGRN rate in Germany (31), resulted in a low number of resistant isolates, which in turn adds a large statistical imprecision to the calculation of VMEs. For future studies, the inclusion of a set of spiked BCs with pathogens of higher resistance rates or MICs around the susceptibility breakpoint of critical antibiotics, e.g., piperacillin/tazobactam or meropenem, would be useful. Notably, previous studies using the FAST system for the analysis of MDRGN bacteria in spiked BCs did not demonstrate significant differences in categorical agreement (CA) compared with prospective BCs (19, 20, 23, 26). Another limitation is that our SLP, i.e., VITEK 2 AST from short-term culture, is not a widely adopted practice. However, the SLP was internally validated by us and showed an overall performance within the limits required by the CLSI (13).

In 2024, commercialization of the FAST system was discontinued, and the system is no longer available at the time of publication of this article. Nevertheless, the workflow investigated in our study is not specific to the FAST system. It should, in principle, be applicable to any sample-preparation method capable of generating viable bacterial cells from positive BC broth. Consequently, our results are not restricted to the FAST system and may also be relevant to similar technologies.

In summary, the use of the FAST system in combination with MALDI-TOF-MS ID, the β-LACTA test, and VITEK 2 AST demonstrated very good performance. The quality of ID and AST derived from LC samples are suitable for use in routine diagnostics, although they were slightly inferior to SLP. The β-LACTA test detected Enterobacterales expressing ESBL but not AmpC with high sensitivity and specificity within min. VITEK 2 AST was able to deliver same-day results for a considerable proportion of BCs and antibiotics that were positive at laboratory opening and until noon but not for those becoming positive in the afternoon. As a result, the FAST system was able to significantly reduce the time to result compared with SLP. However, in settings with low resistance rates, the impact of rapid AST from positive blood cultures on escalation strategies is generally limited, as empirical therapy is often already effective, but it may offer additional benefits for antimicrobial stewardship by enabling earlier de-escalation.

## Data Availability

The data sets used and/or analyzed during the current study are available from the corresponding author upon reasonable request.

## SUPPLEMENTAL MATERIAL

Supplemental material spectrum.01328-26-s0001.docx Tables S1 to S6.

ASM does not own the copyrights to Supplemental Material that may be linked to, or accessed through, an article. The authors have granted ASM a non-exclusive, world-wide license to publish the Supplemental Material files. Please contact the corresponding author directly for reuse.
