# Supplementary material for: Performance of the Qvella FAST system with downstream application of MALDI-TOF-MS, VITEK 2, and β-LACTA test for same-day identification and antimicrobial susceptibility testing of Gram-negative rods directly from positive blood cultures
Source: Microbiol Spectr. 2026 Jul 14;14(8):e01328-26. doi: 10.1128/spectrum.01328-26 (PMC13436139; doi:10.1128/spectrum.01328-26)
Supplement: Supplementary file 1 [file spectrum.01328-26-s0001.docx]

**Supplemental materials for manuscript by Esse *et al.*, “Performance of the Qvella FAST™ system with downstream application of MALDI-TOF-MS, VITEK^®^ 2, and β-LACTA™ test for same-day identification and antimicrobial susceptibility testing of Gram-negative rods directly from positive blood cultures”**

Supplementary Table 1: Review of the literature for the FAST™ system with focus on Gram-negative bacteria

| **Author** | **Year**  **Country** | **No. of BC**  [prospective/ spiked]  (total no. of GN bacteria) | **System**  **failure**  (total no. of runs) | **ID-agreement^a^ on genus level**  (no. of GN bacteria) | **Reference**  **AST-System** | **Categorical agreement***  (no. of GN bacteria) | **Essential agreement^a^**  (no. of GN bacteria) | **Other tests**  (no. of GN bacteria;  tested material) | **Clinical impact^b^**  (no. of GN bacteria) |
| --- | --- | --- | --- | --- | --- | --- | --- | --- | --- |
| Verroken  *et al.* (20) | 2022  Belgium | 198/80  (124) | 0.4%  (278) | 95.2%  (124) | BD Phoenix™ (*Enterobacterales*)  DDT  (*Pseudomonas* spp.) | 97.8%  (*Enterobacterales*; 73)  99.0%  (*Pseudomonas* spp; 25) | n.d. | β-Lacta™  (66; LC);  sensitivity 95.8% | n.d. |
| Ugaban  *et al.* (23) | 2022  USA | 131/23  (64) | 2.1%  (142) | 90.6%  (64) | VITEK® 2 | 98.0% (47) | 98.4% | Verigene®  (51; BC) | n.d. |
| Grinberg  *et al.* (24) | 2022  Germany | 250/0  (80) | 7.2%  (250) | 92.5%  (80) | VITEK® 2 | 98.5% (70) | n.d. | none | n.d. |
| Kuo  *et al.* (19) | 2022  USA | 309/54  (132) | 16.5%  (363) | 95.5%  (132) | BD Phoenix™ | 97.8% (120) | 98.5% | Verigene®  (80; BC) | n.d. |
| Bonaiuto  *et al.* (22) | 2023  Italy | 241/0  (122) | n.d. | 93.8%  (122) | BMD | 99.1% (122) | 98.2% | OKNVI RESIST‑5  (83; LC) | n.d. |
| Maddalena *et al.* (21) | 2023  Italy | 100/0  (48) | n.d. | 100.0%  (48) | BD Phoenix™ | 100.0% (48) | 100.0% | none | n.d. |
| Sy  *et al.* (25) | 2023  Germany | 261/0  (70) | 2.7%  (261) | 95.7%  (70) | MicroScan WalkAway *plus* | 97.3%  (*Enterobacterales*; 54)  78.4%  (*Pseudomonas* spp.; 4) | n.d. | none | n.d. |
| Penven  *et al*. (26) | 2024 France | 107/102  (110) | 1.4%  (209) | 90.9%  (110) | DDT | 99.56% (2,692 drug-bacteria combinations) | n.d. | manual work-flow (110) | n.d. |
| Esse *et al.* (this study) | 2026  Germany | 109/0  (109)^c^ | 3.4%  (119) | 97.2%  (109) | VITEK® 2 | 99.3% (99)^d^ | 99.2% (99)^d^ | β-Lacta™  (91; LC)^e^; sensitivity 87.5% | 7.4%  (94)^f^ |

^a^Reference ID system in all studies was MALDI Biotyper®; results shown for analysis of Gram-negative bacteria only; ^b^clinical impact is defined as earlier detection of ineffective empiric antimicrobial therapy; ^c^after exclusion of ten samples due to mixed cultural growth (n=6) and system failure (n=4); ^d^after exclusion of ten samples due to disc-diffusion-testing (n=8) or classification as contamination (clinically or during laboratory handling, n=2); ^e^after exclusion of eight samples because non-Enterobacterales were detected; ^f^after exclusion of five samples due to missing data on empiric therapy (n=4) or the patient died prior to completion of the test run (n=1). No., number; BC, blood-culture; GN, Gram-negative; ID, identification; AST, antibiotic susceptibility testing; DDT, disc diffusion test; LC, liquid colony; n.d. not determined; BMD, broth microdilution

Supplementary Table 2: MALDI-TOF-MS results from Liquid Colony™ with MALDI-TOF-MS score below 2.0

| **Study no.** | **MALDI-TOF-MS score from LC (reliability)** | **ID of best MALDI-TOF-MS score** | **No. of top 10 IDs with similar ID result** | **Similar ID within 10 best matching IDs** | **MALDI-TOF-MS score from SLP** | **ID of best MALDI-TOF-MS score from SLP** |
| --- | --- | --- | --- | --- | --- | --- |
| 59 | 1.40 (low) | *Capnocytophaga sputigena* | 1 | 6 | 2.50 | *Capnocytophaga sputigena* |
| 107 | 1.48 (low) | *Escherichia coli* | 6 | 7 | 2.20 | *Escherichia coli* |
| 118 | 1.68 (low) | *Burkholderia gladioli* | 1 | 5 | 2.22 | *Burkholderia gladioli* |
| 21 | 1.76 (medium) | *Enterobacter cloacae* complex | 7 | 9 | 2.29 | *Enterobacter cloacae* complex |
| 20 | 1.78 (medium) | *Acinetobacter ursingii* | 10 | 10 | 2.05 | *Acinetobacter ursingii* |
| 67 | 1.79 (medium) | *Bacteroides ovatus* | 6 | 6 | 2.02 | *Bacteroides ovatus* |
| 92 | 1.90 (medium) | *Escherichia coli* | 7 | 8 | 2.44 | *Escherichia coli* |
| 41 | 1.96 (medium) | *Stenotrophomonas maltophilia* | 10 | 10 | 2.21 | *Stenotrophomonas maltophilia* |
| 2 | 1.92 (medium) | *Escherichia coli* | 1 | 6 | 2.32 | *Escherichia coli* |
| 95 | 1.92 (medium) | *Escherichia coli* | 10 | 10 | 2.26 | *Escherichia coli* |
| 106 | 1.93 (medium) | *Escherichia coli* | 10 | 10 | 2.56 | *Escherichia coli* |
| 85 | 1.94 (medium) | *Pseudomonas putida* complex | 8 | 8 | 2.27 | *Pseudomonas putida* complex |

No., number; MALDI-TOF-MS, matrix-assisted laser desorption/ionization time-of-flight mass spectrometry; LC, Liquid Colony™; ID, identification; SLP, standard laboratory procedure.

Supplementary Table 3: Percentage of VITEK® 2 AST results from Liquid Colony™ available after six hours of incubation

| **Antibiotic** | **VITEK® 2 AST results from LC available after 6 h [%]** |
| --- | --- |
| **Ampicillin/sulbactam** | 94.5 |
| **Tigecycline** | 94.5 |
| **Moxifloxacin** | 93.4 |
| **Ciprofloxacin** | 91.7 |
| **Gentamicin** | 90.6 |
| **Tobramycin** | 90.6 |
| **Cefotaxime** | 83.5 |
| **Ampicillin** | 78.0 |
| **Ceftazidime** | 76.0 |
| **Cefuroxime** | 74.7 |
| **Cotrimoxazole** | 47.3 |
| **Imipenem** | 46.9 |
| **Meropenem** | 0 |
| **Piperacillin/tazobactam** | 0 |

AST, antimicrobial susceptibility testing; LC, Liquid Colony™

Supplementary Table 4: MIC deviation of VITEK® 2 AST between Standard Laboratory Procedure and Liquid Colony™

| **MIC deviation** | **≤ -3** | **-2** | **-1** | **0** | **+1** | **+2** | **≥ +3** |
| --- | --- | --- | --- | --- | --- | --- | --- |
| **Total**  [no. of deviations/no. tested isolates] (%) | 2/1279  (0.2) | 0/1279  (0.0) | 38/1279  (3.0) | 1209/1279  (94.5) | 22/1279  (1.7) | 2/1279  (0.2) | 6/1279  (0.5) |
| **Ampicillin**  [no. of deviations/no. tested isolates] (%) | 0/91  (0.0) | 0/91  (0.0) | 6/91  (6.6) | 82/91  (90.1) | 3/91  (3.3) | 0/91  (0.0) | 0/91  (0.0) |
| **Ampicillin/sulbactam**  [no. of deviations/no. tested isolates] (%) | 0/91  (0.0) | 0/91  (0.0) | 16/91  (17.6) | 70/91  (76.9) | 4/91  (4.4) | 1/91  (1.1) | 0/91  (0.0) |
| **Piperacillin/tazobactam***  [no. of deviations/no. tested isolates] (%) | 1/97  (1.0) | 0/97  (0.0) | 1/97  (1.1) | 90/97  (92.8) | 3/97  (3.1) | 0/97  (0.0) | 2/97  (2.1) |
| **Cefuroxime^a^**  [no. of deviations/no. tested isolates] (%) | 0/80  (0.0) | 0/80  (0.0) | 3/80  (3.7) | 75/80  (93.8) | 2/80  (2.5) | 0/80  (0.0) | 0/80  (0.0) |
| **Cefotaxime**  [no. of deviations/no. tested isolates] (%) | 0/91  (0.0) | 0/91  (0.0) | 0/91  (0.0) | 87/91  (95.6) | 2/91  (2.2) | 0/91  (0.0) | 2/91  (2.2) |
| **Ceftazidime**  [no. of deviations/no. tested isolates] (%) | 0/99  (0.0) | 0/99  (0.0) | 0/99  (0.0) | 96/99  (97.0) | 2/99  (2.0) | 0/99  (0.0) | 1/99  (1.0) |
| **Meropenem^#^**  [no. of deviations/no. tested isolates] (%) | 0/98  (0.0) | 0/98  (0.0) | 0/98  (0.0) | 96/98  (98.0) | 1/98  (1.0) | 1/98  (1.0) | 0/98  (0.0) |
| **Gentamicin**  [no. of deviations/no. tested isolates] (%) | 0/91  (0.0) | 0/91  (0.0) | 0/91  (0.0) | 91/91  (100) | 0/91  (0.0) | 0/91  (0.0) | 0/91  (0.0) |
| **Tobramycin**  [no. of deviations/no. tested isolates] (%) | 0/99  (0.0) | 0/99  (0.0) | 0/99  (0.0) | 96/99  (97.0) | 3/99  (3.0) | 0/99  (0.0) | 0/99  (0.0) |
| **Ciprofloxacin**  [no. of deviations/no. tested isolates] (%) | 0/99  (0.0) | 0/99  (0.0) | 4/99  (4.0) | 93/99  (93.9) | 1/99  (1.0) | 0/99  (0.0) | 1/99  (1.0) |
| **Moxifloxacin**  [no. of deviations/no. tested isolates] (%) | 0/91  (0.0) | 0/91  (0.0) | 2/91  (2.2) | 88/91  (96.7) | 1/91  (1.1) | 0/91  (0.0) | 0/91  (0.0) |
| **Trimethoprim/ sulfamethoxazole**  [no. of deviations/no. tested isolates] (%) | 0/91  (0.0) | 0/91  (0.0) | 1/91  (1.1) | 90/91  (98.9) | 0/91  (0.0) | 0/91  (0.0) | 0/91  (0.0) |
| **Tigecycline^b^**  [no. of deviations/no. tested isolates] (%) | 0/62  (0.0) | 0/62  (0.0) | 0/62  (0.0) | 62/62  (100) | 0/62  (0.0) | 0/62  (0.0) | 0/62  (0.0) |
| **Imipenem**  [no. of deviations/no. tested isolates] (%) | 1/99  (1.0) | 0/99  (0.0) | 5/99  (5.1) | 93/99  (93.9) | 0/99  (0.0) | 0/99  (0.0) | 0/99  (0.0) |

MIC deviation between VITEK® 2 of LC and SLP. Deviation between -1 and +1 MIC dilution (grey background) are defined as fulfilled essential agreement. *Two measurement errors; ^#^one measurement error; ^a^EUCAST breakpoints for cefuroxime only applicable for *Escherichia coli*, *Klebsiella* spp. (except for *Klebsiella aerogenes*), *Raoultella* spp. and *Proteus mirabilis*; ^b^EUCAST breakpoints for tigecycline only applicable for *Escherichia coli* and *Citrobacter koseri*; No., number.

Supplementary Table 5: Cross table of the β-LACTA™ test performed with samples from the Liquid Colony™

|  | | **Third-generation cephalosporin resistance in SLP** | | **Total** |
| --- | --- | --- | --- | --- |
|  |  | positive | negative |  |
| **β-LACTA™** | Positive | 14 | 1^b^ | 15 |
|  | Negative | 2^a^ | 74 | 76 |
| **Total** | | 16 | 75 | 91 |

^a^false-negative results occurred in *Enterobacter cloacae* complex (n=1) and *Klebsiella aerogenes* (n=1);
^b^false-positive result occurred in *Klebsiella oxytoca* (n=1). Sensitivity=87.5%, specificity=98.7%.

Supplementary Table 6: Analysis of false-negative β-LACTA™ test results

| **Study no.** | **Pathogen** | **β-LACTA™ test result** | **eazyplex® BloodScreen GN result** | **Cefotaxime MIC VITEK® 2 from LC (mg/l)** | **Ceftazidime MIC VITEK® 2 from LC (mg/l)** | **Piperacillin/tazobactam MIC VITEK® 2 from LC (mg/l)** | **Meropenem MIC VITEK® 2 from LC (mg/l)** |
| --- | --- | --- | --- | --- | --- | --- | --- |
| 14 | *Enterobacter cloacae* complex | negative | CTX-M-1 group: negative  CTX-M-9 group: negative | ≥ 64 | ≥ 64 | ≥ 128 | ≤ 0,25 |
| 51 | *Klebsiella aerogenes* | negative | CTX-M-1 group: negative  CTX-M-9 group: negative | 8 | 16 | ≥ 128 | ≤ 0,25 |

No., number; MIC, minimum inhibitory concentration; LC, liquid colony™.

Calculations used for the rate of VME, ME, mE:

VME = (no. of VME of an antibiotic / total no. of true resistant results of the antibiotic) x 100

ME = (no. of ME of an antibiotic / total no. of true susceptible results of the antibiotic) x 100

mE = (no. mE of an antibiotic / total no. of results of the antibiotic) x 100
